# Evaluation of disparities in the incidence, presentation, and treatment of pediatric differentiated thyroid cancer in the United States (2000–2022)

**DOI:** 10.1371/journal.pone.0333401

**Published:** 2025-10-16

**Authors:** Xuejuan Ning, Rong Wang, Jennifer Ogilvie, Catherine Dinauer, Xiaomei Ma, Nicole C. Deziel

**Affiliations:** 1 Department of Environmental Health Sciences, Yale School of Public Health, New Haven, Connecticut, United States of America; 2 Department of Chronic Disease Epidemiology, Yale School of Public Health, New Haven, Connecticut, United States of America; 3 Department of Surgery (Endocrine Surgery), Yale School of Medicine, New Haven, Connecticut, United States of America; 4 Department of Pediatrics (Endocrinology), Yale School of Medicine, New Haven, Connecticut, United States of America; 5 Yale Comprehensive Cancer Center, Yale School of Medicine, New Haven, Connecticut, United States of America; Cincinnati Children’s Hospital Medical Center, UNITED STATES OF AMERICA

## Abstract

**Introduction:**

Rates of pediatric differentiated thyroid cancer (DTC) have been increasing, yet disparities in incidence, diagnosis, and treatment across race and ethnicity have not been fully explored.

**Methods:**

We assessed temporal trends in the incidence of pediatric DTC using data from 2000–2022 (excluding 2020 due to COVID-19) from the National Childhood Cancer Registry. Annual percent changes (APC) were calculated using joinpoint regression analysis overall and by race, ethnicity, age, and clinical factors. Differences in cancer presentation and treatment by race and ethnicity were evaluated using data from Surveillance, Epidemiology, and End Results (SEER) for 18 registries for 2006–2018, while accounting for socioeconomic status (SES).

**Results:**

Overall pediatric DTC incidence increased 4.5% annually from 2000−2018 (95%CI: 3.8%−5.8%), then declined (−6.9%) through 2022 (95%CI: −14.5%, −1.2%). Rates of decline appeared sharpest among Non-Hispanic White patients. Incidence rates continued increasing among Non-Hispanic Asian/Pacific Islanders and patients diagnosed with tumors >4 cm. Compared to non-Hispanic White patients, non-Hispanic Black, non-Hispanic Asian/Pacific Islander, and Hispanic children were more likely to be diagnosed with a tumor >4 cm.

**Conclusion:**

Declines in reported pediatric thyroid cancer incidence, particularly among the smallest tumor sizes, after 2018 may be attributable to application of thyroid cancer management guidelines. However, the continued increase among those presenting with larger tumor sizes may support a true continued increase in incidence among some groups. The greater proportion of non-White children being diagnosed with larger tumors could be due to inequities related to timely access to care, differential application of thyroid management guidelines, differences in cancer subtypes, or other factors. These findings warrant further exploration when additional years of data are available.

## Introduction

Pediatric thyroid cancer affects approximately 5 children per million in the United States (US) [1]. The papillary and follicular subtypes account for approximately 87% and 8% of all pediatric thyroid cancer cases, respectively [2], and together are classified as differentiated thyroid cancers (DTC). Compared to adult cases, pediatric DTC tends to be more extensive at diagnosis and present at later stages, yet has an extremely low mortality rate [3–6]. Despite the excellent prognosis, pediatric DTC survivors may experience secondary effects of therapy, notably increased rates of leukemia and salivary gland cancer in those treated with radioactive iodine [7]; physical problems and fatigue [8]; disruptions to life milestones (e.g., education, employment, family formation); or mental health conditions (e.g., anxiety, depression) [9–11]. Disparities in incidence rates, presentation, and prognosis have been observed by social factors such as race and ethnicity [12–19] and socioeconomic status [12,20] as well as biological factors such as age of diagnosis [21,22].

Pediatric DTC incidence rates have been increasing across multiple racial and ethnic groups, as documented by average annual percent changes (APC). Reported APCs from 1998–2013 were documented 4% in non-Hispanic White, 3% in non-Hispanic Black, and 5% in Hispanic children [23,24]. Rising incidence rates have been observed in adolescent age groups (APC of 5% in 10–14 year olds and 4% in 15–19 year olds) as well as in younger age groups, albeit with smaller magnitude of increase (APC: 0.8% in 0–9 year olds) [23]. Pediatric DTC incidence rates increased up to the 2010s [2,25,26], but an apparent plateau or decline began in 2014 [27,28]. Whether or not the decrease has continued in subsequent years or has exhibited a similar rate of decline across different population subgroups has not yet been established. Identification of populations at greater risk of pediatric DTC and clarification of differences in temporal trends across different subgroups can inform detection strategies, clinical practices, and epidemiologic research.

Differences in DTC presentation and prognosis have been observed across population subgroups. The mortality rate is higher in those children with DTC who present with distant metastases at diagnosis [29–32]. In addition, limited research suggests that there are racial disparities in pediatric thyroid cancer presentation and prognosis [2,12,13,33,34]. Patients of non-White race and lower socioeconomic status (SES) tend to present at later stages [12,13]. have larger tumors [12], and have poorer survival [2,34,35]. To our knowledge, most studies of disparities in pediatric thyroid cancer have focused on White versus Black or Hispanic patients [2,12,13,24,34] with limited exploration of disparities in less represented racial or ethnic groups, such as Non-Hispanic Asian or Pacific Islanders, who have higher incidence and worse cancer presentations than non-Hispanic Whites among adult patients [15,16]. Also, prior studies on social determinants of pediatric thyroid cancer outcomes in the national population generally considered SES factors at relatively large spatial scales (e.g., county-level) [13,36]; few studies accounted for SES variables at finer spatial resolution [12,37]. As racial and ethnic disparities and socioeconomic status are often intertwined, it would be crucial to control for finer SES when investigating racial and ethnic disparities and cancer-related outcomes.

To further investigate recent pediatric DTC trends and disparities, we carried out this analysis with the following objectives: (1) describe temporal trends in DTC incidence in pediatric patients using the most recent data available (2000–2022) across a range of population subgroups; and (2) examine disparities among several racial and ethnic categories in cancer presentation and treatment while accounting for census tract level SES in the US population.

## Methods

### Data sources

For our first objective of evaluating temporal trends in DTC incidence by population subgroups, we obtained a dataset from the population-based registries of the Surveillance, Epidemiology, and End Results Program (SEER), a program supported and maintained by the National Cancer Institute to provide national representative cancer statistics to reduce cancer burden. The dataset “Incidence – SEER Research Data with Delay-Adjustment, 17 Registries, Malignant Only, Nov 2024 Sub (2000-2022)” was used and covers cancer cases from 17 registries, which approximately covers 26.5% of the US population. The registries are San Francisco-Oakland Standard Metropolitan Statistical Areas (SMSA), Connecticut, Hawaii, Iowa, New Mexico, Seattle (Puget Sound), Utah, Atlanta (Metropolitan), San Jose-Monterey, Los Angeles, Alaska Natives, Rural Georgia, California excluding San Francisco/San Jose-Monterey/Los Angeles, Kentucky, Louisiana, New Jersey, and Greater Georgia. Pediatric patients (<20 years old) with a DTC diagnosis from 2000−2019, 2021, and 2022 were identified and included for the temporal trend analyses. We removed data of year 2020 for incidence rate temporal trend analysis because COVID-19 pandemic might have decreased health care utilization [38]. We selected this dataset because it had the most recent data coverage and corrected for the lag in reporting diagnosis while also providing more detailed information on race, ethnicity, age, and thyroid cancer subtypes. Hereafter we refer to this as the SEER-17 database.

Data for our second objective focused on social and demographic factors related to DTC presentation and treatment used the SEER database – “Incidence – SEER Research Plus Data (Specialized with Census Tract SES/Rurality), 18 registries (excl AK), Nov 2020 Sub (2006-2018)”. This database covers approximately 28% of the US population and includes cancer records from registries in San Francisco, Connecticut, Detroit, Hawaii, Iowa, New Mexico, Seattle, Utah, Atlanta, San Jose-Monterey, Los Angeles, Rural Georgia, California excluding San Francisco/San Jose-Monterey/Los Angeles, Kentucky, Louisiana, New Jersey, and Greater Georgia [39]. This dataset was selected due to the availability of harmonized census-tract level SES variables, which were available through 2018. Due to confidentiality concerns, there were no geographic identifiers in this database. Pediatric patients (<20 years old) with a primary DTC diagnosis and non-missing harmonized SES variables from 2006−2018 were included. Hereafter, we refer to this as the SEER-18 database.

### Case ascertainment and tumor characteristics

In both databases, DTC cases were those classified by the International Classification of Diseases for Oncology, 3rd edition codes as papillary (ICD-O-3: 8050, 8052, 8130, 8260, 8340–8344, 8350, 8450, and 8452) or follicular (ICD-O-3: 8290, 8330–8332, and 8335). Three tumor staging schemes were used across our study period in the NCCR database: SEER summary stage 1977 (1995–2000), SEER summary stage 2000 (2001–2003), and Summary Stage (2004+). Summary/Historic. Combined Summary Stage (2004+) was used to determine tumor staging in the SEER-18 database. In both databases, we categorized tumor staging as localized (“in situ” or “localized only”), regional (“regional by direct extension only”, “regional lymph nodes involved only”, “regional by both direct extension and lymph node involvement”, and “regional NOS”), distant (“distant site(s)/node(s) involved”), or unknown. Tumor size was recorded using 3 schemes in SEER-17 database: Historic Extent of Disease 10 (2000–2003), Collaborative Staging codes (2004–2016) and Tumor size summary (2016+). The latter two schemes were used to define tumor size in SEER-18 database. Because tumor size coding was not available for the years 1997–2003, the tumor size data for that period was missing. We harmonized these schemes to derive a tumor size of ≤ 2 cm, > 2–4 cm and larger than 4 cm since tumors ≤ 2 cm are considered as small tumors and tumors > 4 cm are correlated with increased risk of metastasis [40]. Radiation treatment was classified as none, use of radioisotopes (e.g., radioactive iodine), and other radiation (e.g., beam radiation).

### Demographic characteristics

For both databases, individual-level demographic characteristics obtained included age at diagnosis, sex, and race and ethnicity. Age was grouped into 0–9, 10–14, and 15–19 years old to facilitate comparisons with other literature. Race and ethnicity variables were categorized as Non-Hispanic White, Non-Hispanic Black, Hispanic, Non-Hispanic Asian/Pacific Islander, Non-Hispanic American Indian or Alaska Native and Other for SEER-17. For SEER-18, race and ethnicity variables were categorized as Non-Hispanic White, Non-Hispanic Black, Hispanic, Non-Hispanic Asian/Pacific Islander, and Non-Hispanic Other (including Non-Hispanic American Indian and Alaska Native or Unknown).

In SEER-18, community-level variables representing SES at the Census tract level included the Yost index, which is a composite score available in five quintiles that reflects seven different SES constructs: household income, median house value, median rent, percent below 150% of poverty line, education index, percent working class, and percent unemployed. We also obtained a variable for persistent poverty, which identifies census tracts with 20% or more of the population having lived below the poverty line for 30 years (yes/no). We acquired a variable for urbanicity/rurality, which is determined based on the Census Bureau’s four-category variable that reports the percent of the population living in non-urban areas (urban/rural).

### Statistical analysis

To address our first objective, we examined the incidence rate of pediatric DTC during the study period (2000–2022) and temporal changes of incidence rate by sociodemographic factors including sex, race/ethnicity, and age group. We considered these by cancer-related factors such as histologic type, tumor stage, and tumor size using descriptive statistics. SEER*Stat, version 8.4.5 (National Cancer Institute; Information Management Services) was used to calculate incidence rate and incidence rate temporal trends. The incidence rate was standardized by the 2000 US standard population and was reported as per 1,000,000 person-years. Joinpoint Regression Program, version 5.4.0 was used to generate incidence temporal trend and calculate the annual percentage change (APC) and its 95% confidence intervals (CI). The best fit model with the optimal number of joints was determined using the Weighted BIC method, and the Empirical Quantile (EmpQ) method was used to produce a more accurate confidence interval.

For our second objective, we investigated racial and SES disparities in cancer presentation and treatment using logistic regression models and linear regression models. Cancer presentation and treatment outcomes were advanced stage at diagnosis (distant vs. localized/regional), large tumor size (>4 cm vs. ≤ 4 cm), receiving less than a total thyroidectomy (yes/no), and receiving radiation therapy (yes/no). We conducted univariable and multivariable analyses. Multivariable models for advanced stage at diagnosis and larger tumor at diagnosis were adjusted for age, sex, SES quintile, rural/urban, persistent poverty, and cancer subtype (papillary vs. follicular). The multivariable models for receiving less than a total thyroidectomy and receiving radiation therapy were further adjusted for tumor size and tumor stage. In addition, we tested child age (0–14 vs. 15–19 years old) as an effect modifier by adding race/ethnicity group and age interaction terms in the adjusted models. All analyses were conducted using R Version 4.1.1. We considered a two-tailed p < 0.05 as the statistically significant threshold.

## Results

### Incidence analysis

A total of 4,189 pediatric DTC cases were diagnosed during 2000–2022 (excluding 2020) and therefore included in the incidence analysis (**Table 1**). Cases were mostly female (3,408 [81%]), 15–19 years old (3,186 [76%]), and Non-Hispanic White (2,310 [55%]). The most common histologic type was papillary (92%), with a small proportion having follicular thyroid cancer (8%). The malignancies were primarily localized (47%) or regional (48%) and tumors were most commonly ≤ 2 cm (45%) or >2–4 cm (33%). The incidence rates for the study period were 4 times higher in females (14.0/10^6^) than males (3.0/10^6^). The incidence rate in children aged 15–19 years (25.2/10^6^) was 4 times that of 10–14-year-olds (6.8/10^6^); 0–9-year-olds had the lowest incidence rate (0.6/10^6^). Incidence rate was the highest in racial and ethnic group reported as Non-Hispanic White (9.8/10^6^), closely followed by Non-Hispanic Asian/Pacific Islander (9.5/10^6^), Hispanic (8.1/10^6^), Non-Hispanic American Indian or Alaska Native (6.4/10^6^), and Non-Hispanic Black (2.7/10^6^).

**Table 1:** Patient characteristics and incidence rates of differentiated pediatric thyroid cancer, SEER-17 (2000-2019, 2021-2022).

	N (%)	Age and delay-adjusted incidence rate, per 1,000,000 person-years (95%CI)
Overall	4189 (100)	8.4 (8.1, 8.6)
Sex		
Male	781 (19)	3.0 (2.8, 3.3)
Female	3408 (81)	14.0 (13.5, 14.5)
Age, years		
0-9	142 (3)	0.6 (0.5, 0.7)
10-14	861 (21)	6.8 (6.4, 7.3)
15-19	3186 (76)	25.2 (24.4, 26.1)
Race/ethnicity		
Non-Hispanic White	2310 (55)	9.8 (9.4, 10.2)
Non-Hispanic Black	174 (4)	2.7 (2.3, 3.1)
Hispanic	1189 (28)	8.1 (7.7, 8.6)
Non-Hispanic Asian/Pacific Islander	431 (10)	9.5 (8.6, 10.4)
Non-Hispanic American Indian or Alaska Native	26 (1)	6.4 (4.2, 9.4)
Other	59 (1)	NA
Histologic type		
Papillary	3846 (92)	7.7 (7.5, 7.9)
Follicular	343 (8)	0.7 (0.6, 0.8)
Stage		
Localized	1950 (47)	3.9 (3.7, 4.1)
Regional	2017 (48)	4.0 (3.9, 4.2)
Distant	155 (4)	0.3 (0.3, 0.4)
Unknown	67 (1)	0.1 (0.1, 0.2)
Tumor size, cm		
≤ 2	1901 (45)	3.8 (3.6, 4.0)
> 2-4	1394 (33)	2.8 (2.6, 2.9)
> 4	628 (16)	1.3 (1.2, 1.4)
Unknown	266 (6)	0.5 (0.5, 0.6)

In terms of the trends over time, the APC in overall pediatric DTC per 1,000,000 person-years increased an average of 2.3% per year (95%CI: 1.4%, 3.3%) over the full study period (**Table 2**). Pediatric DTC rates increased across most population strata. The average APC for males was slightly lower (2.2% [95%CI: −0.1%, 6.8%]) compared to females (2.9% [95%CI:1.3%, 5.3%]). When considering race and ethnicity, greater increases were observed for Non-Hispanic Asian/Pacific Islander (4.5% [95%CI: 2.9%, 6.8%]) and Hispanic (4.0% [95%CI: 2.3%, 7.5%]) children compared to the other groups. Among age groups, individuals 15–19 years old (3.1% [95%CI: 2.0%, 4.3%]) had a significant increase; increases in average APC were not observed among 10–14 years (1.6% [95%CI: −0.2%, 4.4%]) and 0–9 years age group (0.8% [95%CI: −2.0%, 4.0%]). For histological types, there was an increase for the average APC of papillary thyroid cancer (2.7% [95%CI: 1.7%, 3.7%]) but not follicular (−0.2% [95%CI: −2.9%, 2.5%]). Among tumor stage groups, the largest average increase in APC was in regional staged tumors (3.6% [95%CI: 2.5%, 4.9%]), followed by localized tumors (2.1% [95%CI: 0.7%, 3.7%]); an increase was not observed for distant tumors (0.7% [95%CI: −2.0%, 3.6%]). APC of tumors of all sizes on average increased with the largest increase for tumors >4 cm (4.2% [95%CI: 2.4%, 6.5%]), followed by tumors of >2–4 cm (2.6% [95%CI: 1.2%, 5.5%]) and tumors ≤ 2 cm (2.7% [95%CI: 1.7%, 3.6%]).

**Table 2:** Patient incidence temporal trends by demographic factors and tumor characteristics, SEER-17 (2000-2019, 2021).

	Overall	Trend 1		Trend 2^		Trend 3^			Trend 4^	
Characteristics	AAPC, % (95%CI)	Years	APC, % (95%CI)	Years	APC, % (95%CI)	Years	APC, % (95%CI)	Years		APC, % (95%CI)
Overall	2.3 (1.4, 3.3)	2000-2018	4.5 (3.8, 5.8)	2018-2022	−6.9 (−14.5, −1.2)	–	–	–		–
Sex										
Male	2.2 (−0.1, 6.8)	2000-2018	4.6 (2.1, 43.5)	2018-2022	−7.8 (−23.5, 3.9)	–	–	–		–
Female	2.9 (1.3, 5.3)	2000-2015	5.3 (3.9, 16.5)	2015-2022	−2.0 (−10.9, 2.3)	–	–	–		–
Race/ethnicity*										
Non-Hispanic White	1.1 (0.5, 1.7)	2000-2005	−1.0 (−7.2, 2.0)	2005-2011	7.8 (5.5, 13.7)	2011-2019	2.2 (0.2, 3.8)	2019-2022		−10.7 (−14.0, −5.9)
Non-Hispanic Black	2.8 (0.1, 6.2)	2000-2012	10.3 (6.3, 20.7)	2012-2022	−5.5 (−14.1, −0.6)	–	–	–		–
Hispanic	4.0 (2.3, 7.5)	2000-2015	6.7 (4.9, 33.5)	2015-2022	−1.5 (−12.9, 3.6)	–	–	–		–
Non-Hispanic Asian/Pacific Islander	4.5 (2.9, 6.8)	2000-2022	4.5 (2.9, 6.8)	–	–	–	–	–		–
Age, yrs										
0-9	0.8 (−2.0, 4.0)	2000-2022	0.8 (−2.0, 4.0)	–	–	–	–	–		–
10-14	1.6 (−0.2, 4.4)	2000-2019	4.7 (3.4, 9.1)	2019-2022	−16.1 (−27.2, 1.8)	–	–	–		–
15-19	3.1 (2.0, 4.3)	2000-2015	5.3 (4.2, 8.2)	2015-2022	−1.5 (−8.0, 1.9)	–	–	–		–
Histologic type										
Papillary	2.7 (1.7, 3.7)	2000-2018	4.9 (4.1, 6.3)	2018-2022	−6.8 (−14.8, −0.5)	–	–	–		–
Follicular	−0.2 (−2.9, 2.5)	2000-2011	4.7 (1.0, 27.5)	2011-2022	−4.8 (−18.1, −1.0)	–	–	–		–
Stage										
Localized	2.1 (0.7, 3.7)	2000-2015	4.7 (3.4, 8.7)	2015-2022	−3.4 (−11.0, 0.7)	–	–	–		–
Regional	3.6 (2.5, 4.9)	2000-2018	5.8 (4.9, 7.9)	2018-2022	−6.0 (−15.0, 1.2)	–	–			
Distant	0.7 (−2.0, 3.6)	2000-2022	0.7 (−2.0, 3.6)	–	–	–	–	–		–
Tumor size, cm										
≤ 2	2.7 (1.7, 3.6)	2000-2018	5.7 (4.8, 7.0)	2018-2022	−9.9 (−17.8, −4.3)	–	–	–		–
> 2-4	2.6 (1.2, 5.5)	2000-2019	4.0 (1.1, 25.9)	2019-2022	−6.1 (−15.1, 4.3)	–	–	–		–
> 4	4.2 (2.4, 6.5)	2000-2022	4.2 (2.4, 6.5)	–	–	–	–	–		–

^ Subgroups with monotonic trends are marked with “-” for trend 2, 3 and 4.

* Non-Hispanic AIAN and other race/ethnicity groups have incomplete data, hence not presented here.

**Fig 1:**
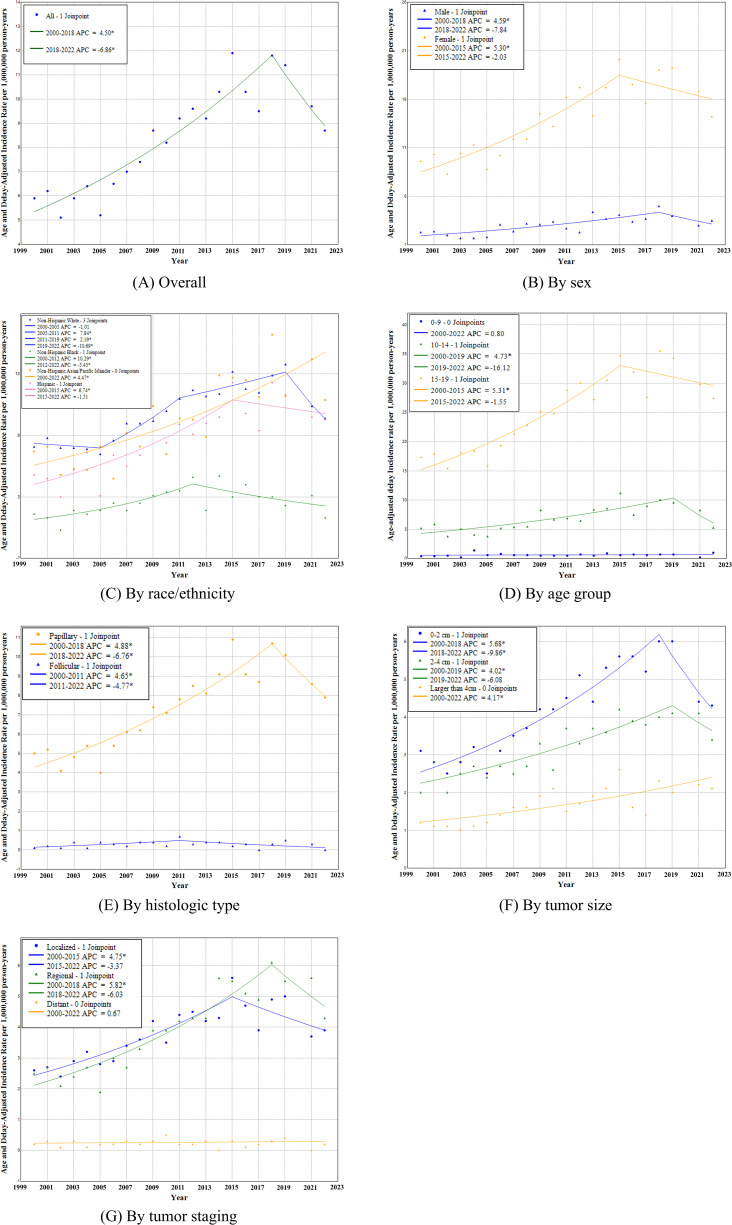
Age and Delay-adjusted pediatric differential thyroid cancer incidence rate trend, 2000–2019, 2021-2022.

A closer look at the temporal trend reveals that while pediatric DTC incidence increased nearly universally from 2000–2018, the overall incidence declined after year 2018 (−6.9% [95%CI: −14.5%, −1.2%]); (**Fig 1**, **Table 2**). However, not all groups experienced a decrease, and the magnitude of the decline varied across groups. A decline was observed in females (−2.0% [95%CI: −10.9%, 2.3%]), males (−7.8% [95%CI: −23.5%, 3.9%]), children aged 10–14 years (−16.1% [95%CI: −27.2%, 1.8%]) and 15–19 years (−1.5% [95%CI: −8.0%, 1.9%]) but not 0–9 years. There was a more pronounced decline among Non-Hispanic White (−10.7% [95%CI: −14.0%, −5.9%]) after year 2019 and in Non-Hispanic Black (−5.5% [95%CI: −14.1%, −0.6%]) after year 2012. For histological types, we observed a decline for papillary (−6.8% [95%CI: −14.8%, −0.5%]) after year 2018 and for follicular type (−4.8% [95%CI: −18.1%, −1.0%]) after year 2011. For tumor stage groups, we observed the largest magnitude of decline in regional tumors (−6.0% [95%CI: −15.0%, 1.2%]) after year 2018, followed by localized tumors (−3.4% [95%CI: −11.0%, 0.7%]). In terms of tumor size, only incidence for tumors ≤2 cm experienced a significant decline (−9.9% [95%CI: −17.8%, −4.3%]), tumors >2–4 cm experienced a smaller magnitude of decline (−6.1% [95%CI: −15.1%, 4.3%]).

### Presentation and treatment analysis

A total of 2,536 patients had harmonized census-tract level SES data in the SEER-18 database, hence were included in the analysis of disparities with thyroid cancer presentations and treatments. Statistically significant differences by race and ethnicity were observed for numerous factors (**Table 3**). Compared to Non-Hispanic White patients, a greater proportion of Non-Hispanic Black patients were diagnosed at the youngest age group of 0–9 years old (4.2% versus 2.7%), while a greater proportion of patients identifying as Non-Hispanic Other were diagnosed at age 15–19 years old (89.1% versus 78.1%) (overall p-value for differences by age = 0.067; **Table 3**). Compared to Non-Hispanic White patients, children of several other race or ethnicities tended to live in census tracts with lower SES as evidenced by differential patterning in the Yost Index (e.g., lowest Yost Index: Non-Hispanic Black: 31.1%; Hispanic: 22.8%; Non-Hispanic Other: 10.6% versus Non-Hispanic White: 8.1%; p < 0.001). Also, Non-Hispanic Black, Hispanic, Non-Hispanic Asian/Pacific Islander and Non-Hispanic Other patients were more likely to live in areas experiencing persistent poverty than Non-Hispanic White patients (19.3%; 13.8%; 4.4%; 8.7% versus 2.8%, respectively; p < 0.001). The differences in patterning in urbanicity/rurality was statistically significant (p < 0.001) with a majority of patients from urban areas and the proportion of rural-residing cases highest among non-Hispanic White children. For cancer presentations, a greater proportion of non-Hispanic Black patients were diagnosed with a localized tumor stage, compared to non-Hispanic White patients (overall p-value for differences by stage <0.001). Greater proportions of Non-Hispanic Black, Hispanic, Non-Hispanic Asian/Pacific Islander and Non-Hispanic Other patients presented with a larger tumor size than Non-Hispanic White patients (e.g., size > 4 cm: 30.3%; 18.9%; 21.6%; 21.7% versus 12.5%) (overall p-value for differences by size<0.001). A larger percentage of Non-Hispanic Black patients were diagnosed with a follicular histology type than other race/ethnicity patients (17.6% versus Non-Hispanic White: 7.9%; Hispanic: 7.2%; Non-Hispanic Asian/Pacific Islander: 7.6%; Non-Hispanic Other: 6.5%). There was some variation in proportion of children receiving a total thyroidectomy with the smallest proportion among Non-Hispanic Black patients (83.2% versus Non-Hispanic White: 87.7%; Hispanic: 90.6%; Non-Hispanic Asian/Pacific Islander: 84.0%; Non-Hispanic Other: 95.7%).

**Table 3:** Patient characteristics by race and ethnicity, census-tract level socioeconomic status and urbanicity/rurality data (2006-2018).

	Non-Hispanic White (n = 1401)	Non-Hispanic Black (n = 119)	Hispanic (n = 720)	Non-Hispanic Asian/Pacific Islander (n = 250)	Non-Hispanic Other (n = 46)	P-value
Sex						0.593
Female	1120 (79.9%)	95 (79.8%)	591 (82.1%)	207 (82.8%)	35 (76.1%)	
Male	281 (20.1%)	24 (20.2%)	129 (17.9%)	43 (17.2%)	11 (23.9%)	
Age, years						0.067
0-9	38 (2.7%)	5 (4.2%)	24 (3.3%)	5 (2.0%)	0 (0%)	
10-14	269 (19.2%)	22 (18.5%)	174 (24.2%)	50 (20.0%)	5 (10.9%)	
15-19	1094 (78.1%)	92 (77.3%)	522 (72.5%)	195 (78.0%)	41 (89.1%)	
Yost index (US-based)						<0.001
Lowest	113 (8.1%)	37 (31.1%)	164 (22.8%)	17 (6.8%)	5 (10.9%)	
Lowest-middle	165 (11.8%)	16 (13.4%)	174 (24.2%)	31 (12.4%)	8 (17.4%)	
Middle	215 (15.3%)	17 (14.3%)	148 (20.6%)	28 (11.2%)	8 (17.4%)	
Middle-high	338 (24.1%)	30 (25.2%)	137 (19.0%)	59 (23.6%)	9 (19.6%)	
Highest	570 (40.7%)	19 (16.0%)	97 (13.5%)	115 (46.0%)	16 (34.8%)	
Persistent poverty						<0.001
Yes	39 (2.8%)	23 (19.3%)	99 (13.8%)	11 (4.4%)	4 (8.7%)	
No	1362 (97.2%)	96 (80.7%)	621 (86.3%)	239 (95.6%)	42 (91.3%)	
Urban/rural						<0.001
Rural	165 (11.8%)	7 (5.9%)	32 (4.4%)	5 (2.0%)	1 (2.2%)	
Urban	1236 (88.2%)	112 (94.1%)	688 (95.6%)	245 (98.0%)	45 (97.8%)	
Tumor stage						<0.001
Localized	692 (49.4%)	79 (66.4%)	293 (40.7%)	104 (41.6%)	18 (39.1%)	
Regional	669 (47.8%)	37 (31.1%)	407 (56.5%)	138 (55.2%)	24 (52.2%)	
Distant	40 (2.9%)	3 (2.5%)	20 (2.8%)	8 (3.2%)	4 (8.7%)	
Thyroid cancer subtype						0.003
Follicular	110 (7.9%)	21 (17.6%)	52 (7.2%)	19 (7.6%)	3 (6.5%)	
Papillary	1291 (92.1%)	98 (82.4%)	668 (92.8%)	231 (92.4%)	43 (93.5%)	
Tumor size						<0.001
≤ 2 cm	761 (54.3%)	41 (34.5%)	302 (41.9%)	117 (46.8%)	19 (41.3%)	
> 2–4 cm	465 (33.2%)	42 (35.3%)	282 (39.2%)	79 (31.6%)	17 (37.0%)	
> 4 cm	175 (12.5%)	36 (30.3%)	136 (18.9%)	54 (21.6%)	10 (21.7%)	
Surgery type						NA
No surgery	1 (0.1%)	0 (0%)	1 (0.1%)	0 (0%)	0 (0%)	
Less than a Lobectomy	8 (0.6%)	0 (0%)	4 (0.6%)	4 (1.6%)	0 (0%)	
Lobectomy and/or isthmectomy	126 (9.0%)	16 (13.4%)	46 (6.4%)	29 (11.6%)	2 (4.3%)	
Subtotal thyroidectomy	34 (2.4%)	4 (3.4%)	16 (2.2%)	7 (2.8%)	0 (0%)	
Total thyroidectomy	1229 (87.7%)	99 (83.2%)	652 (90.6%)	210 (84.0%)	44 (95.7%)	
Surgery (not specified)	3 (0.2%)	0 (0%)	1 (0.1%)	0 (0%)	0 (0%)	
Radiation therapy						0.494
Radioisotopes	796 (56.8%)	75 (63.0%)	431 (59.9%)	138 (55.2%)	30 (65.2%)	
Other radiation	50 (3.6%)	5 (4.2%)	22 (3.1%)	9 (3.6%)	3 (6.5%)	
None	555 (39.6%)	39 (32.8%)	267 (37.1%)	103 (41.2%)	13 (28.3%)	

According to the multivariable models, there was no difference in presenting at an advanced stage of diagnosis by race or ethnicity, except for the Non-Hispanic Other group (**Table 4**). Individuals identifying as Non-Hispanic Black, Hispanic, and Non-Hispanic Asian/Pacific Islander had greater odds of presenting with larger tumor sizes at diagnosis after accounting for demographics, SES factors and histology type, with adjusted OR of 2.57 (95%CI: 1.63, 3.98), 1.54 (95%CI: 1.17, 2.01), and 2.05 (95%CI: 1.44, 2.89) respectively (**Table 4**). The association was stronger in older versus younger Non-Hispanic Black and Hispanic patients; conversely, it was stronger in younger Non-Hispanic Asian/Pacific Islander patients (**Table 5**). As for treatment, Non-Hispanic Asian/Pacific Islanders compared to Non-Hispanic White were 1.41 (95%CI: 0.94, 2.05) times more likely to receive less than a total thyroidectomy, though it was not statistically significant. Also, there was no significant difference in receiving radiation therapy across the race/ethnicity groups.

**Table 4:** Odds ratios and 95% confidence intervals for pediatric differentiated thyroid cancer presentation and initial therapy by race and ethnicity. Racial disparities in pediatric thyroid cancer outcomes, OR/Beta (95%CI).

		Non-Hispanic White	Non-Hispanic Black	Hispanic	Non-Hispanic Asian/Pacific Islander	Non-Hispanic Other
Advanced stage at diagnosis	Crude	1 (Ref)	0.88 (0.21, 2.47)	0.97 (0.55, 1.65)	1.12 (0.48, 2.31)	3.24 (0.94, 8.51)
	Adjusted (1)	1 (Ref)	0.79 (0.18, 2.35)	0.80 (0.43, 1.42)	1.21 (0.51, 2.54)	**3.81 (1.08, 10.46)**
Larger tumor at diagnosis	Crude	1 (Ref)	**3.04 (1.97, 4.60)**	**1.63 (1.28, 2.08)**	**1.93 (1.36, 2.70)**	1.95 (0.90, 3.85)
	Adjusted (1)	1 (Ref)	**2.57 (1.63, 3.98)**	**1.54 (1.17, 2.01)**	**2.05 (1.44, 2.89)**	2.07 (0.95, 4.13)
Receiving less than a total thyroidectomy	Crude	1 (Ref)	1.44 (0.85, 2.35)	0.75 (0.55, 1.00)	1.36 (0.93, 1.96)	0.32 (0.05, 1.06)
	Adjusted (2)	1 (Ref)	1.25 (0.71, 2.13)	0.74 (0.53, 1.02)	1.41 (0.94, 2.05)	0.31 (0.05, 1.05)
Receiving radiation therapy	Crude	1 (Ref)	0.78 (0.52, 1.15)	0.92 (0.76, 1.10)	1.05 (0.80, 1.38)	0.76 (0.40, 1.40)
	Adjusted (2)	1 (Ref)	0.76 (0.50, 1.14)	0.90 (0.74, 1.11)	1.08 (0.82, 1.43)	0.78 (0.41, 1.45)

(1) Adjusted for age, sex, SES quintile, rural/urban, persistent poverty, cancer subtype

(2) Adjusted for age, sex, SES quintile, rural/urban, persistent poverty, cancer subtype, tumor size, stage

**Table 5:** Odds ratios and 95% confidence intervals for pediatric differentiated thyroid cancer presentation and initial management stratified by age, race, and ethnicity.

Outcomes	Age	Non-Hispanic White	Non-Hispanic Black	Hispanic	Non-Hispanic Asian/Pacific Islander	Non-Hispanic Other	P interaction value
Advanced stage at diagnosis (1)	0-14	1 (Ref)	1.21 (0.18, 5.00)	1.04 (0.45, 2.39)	1.54 (0.42, 4.59)	3.80 (0.18, 32.18)	<0.001
	15-19	1 (Ref)	0.61 (0.03, 3.06)	0.59 (0.22, 1.38)	0.99 (0.29, 2.64)	3.65 (0.83, 11.32)	
Larger tumor at diagnosis (1)	0-14	1 (Ref)	1.62 (0.62, 3.96)	1.40 (0.86, 2.28)	2.40 (1.22, 4.62)	1.29 (0.06, 9.24)	0.13
	15-19	1 (Ref)	3.02 (1.79, 4.99)	1.61 (1.16, 2.22)	1.93 (1.27, 2.89)	2.21 (0.96, 4.60)	
Receiving less than a total thyroidectomy (2)	0-14	1 (Ref)	1.49 (0.43, 4.43)	0.42 (0.19, 0.87)	1.63 (0.65, 3.72)	NA	0.58
	15-19	1 (Ref)	1.26 (0.65, 2.30)	0.84 (0.58, 1.21)	1.38 (0.88, 2.11)	0.34 (0.05, 1.19)	
Receiving radiation therapy (2)	0-14	1 (Ref)	0.38 (0.12, 0.99)	0.70 (0.46, 1.07)	2.06 (1.13, 3.79)	NA	<0.001
	15-19	1 (Ref)	0.88 (0.55, 1.38)	0.98 (0.78, 1.24)	0.92 (0.67, 1.26)	0.93 (0.47, 1.77)	

(1) Adjusted for age, sex, SES quintile, rural/urban, persistent poverty, cancer subtype

(2) Adjusted for age, sex, SES quintile, rural/urban, persistent poverty, cancer subtype, tumor size, stage

## Discussion

In the current study, we used data from a nationally representative population from the most recent years available to provide a comprehensive analysis of temporal trends and clinical and patient characteristics related to pediatric thyroid cancer incidence across a range of population subgroups. The data suggest that following a 4.5% per year increase in pediatric DTC from 2000–2018, incidence rates declined significantly overall and among some of the larger population subgroups such as Non-Hispanic White, Non-Hispanic Black, papillary and follicular tumors, and tumors with the smallest tumor sizes (≤2 cm). However, the declines across subgroups were not all statistically significant and were not observed uniformly. In contrast, incidence rates continued rising among children identifying as Non-Hispanic Asian/Pacific Islander, and those with tumor sizes > 4 cm. In addition, we observed some differences in pediatric DTC presentation and treatment among less-studied race and ethnicity groups. We found that compared to Non-Hispanic White, Non-Hispanic Black, Non-Hispanic Asian/Pacific Islander, and Hispanic children were 157%, 105%, and 54%, more likely to be diagnosed with a larger tumor. In addition, we observed a higher proportion of follicular thyroid cancer among non-Hispanic Black patients.

Suggestions of a plateau or decline have been reported previously for subgroups of DTC cases of all ages from SEER population [27,28]. Since the incidence of year 2020 might be heavily impacted by the COVID-19 pandemic as patients were less likely to seek healthcare for non-urgent health conditions [41], we excluded data from 2020, in accordance with SEER guidance. However, the data from 2021 and 2022 still suggests declining trends in several subgroups including male, female, Non-Hispanic White, Non-Hispanic Black, Hispanic, 10–14 years old, 15–19 years old, papillary and follicular histology type, local and regional tumors, and tumors ≤ 4 cm. In contrast, incidence continued to increase throughout the study period among some subgroups, notably cases with larger tumor sizes. It could be the case that healthcare seeking behavior of patients were still influenced by COVID-19 in year 2021–2022. Future research should re-examine the incidence trends using more years of data to see if the declining trend persists. Alternatively, the apparent decline in incidence, particularly among the smaller tumors, may be attributable to changes in clinical practice based on improved awareness of overdiagnosis and possible decrease in nodule biopsies. The American Thyroid Association adult guideline changes in 2009 discouraged biopsy for nodules < 1 cm and the revised guideline published in 2015 recommended higher size threshold for biopsy [40,42–43]. The inaugural American Thyroid Association guideline for children with thyroid nodules and cancer was published in 2015 and advised generally performing Fine Needle Aspiration biopsy for nodules >1 cm but noted that size criteria cannot be as strict in children due to the smaller volume of the thyroid gland [40,43]. Thus, the pediatric guidelines stated that biopsy could be considered in nodules <1 cm if there were suspicious features by ultrasound. Although children were generally less likely to experience overdiagnosis than adults due to a higher threshold for imaging in children [44,45], overdiagnosis still might have played a role in the increasing trend of pediatric thyroid cancer incidence prior to year 2018.

The reason for the continuing rise in incidence of pediatric thyroid cancer cases presenting with tumors size >4 cm could potentially be due to environmental factors or co-morbidities. It has been hypothesized that the increasing rate of obesity among children might have contributed to the parallel trend of rising DTC incidence in adults [46]. A nested case-control study in California found that infants with higher birthweight were more likely to develop pediatric thyroid cancer [47]. Using large prospective cohort studies in Denmark and US, Kitahara et al found child and adult body mass index (BMI) were both associated with elevated risk for adult thyroid cancer, speculating that various hormones may be involved (e.g., estrogen, insulin, leptin, thyroid stimulating hormone, etc.) [46,48,49]. Some have hypothesized that endocrine-disrupting chemicals, such as flame retardants and pesticides might be associated with the risk of thyroid cancer based on evidence in adults [50–53]. A case-control study found that flame retardants, especially decabromodiphenyl ether in dust was associated with higher odds of papillary thyroid cancer in adults [50]. Several studies conducted among pesticide applicators and their spouses showed a link between occupational pesticides exposure and thyroid cancer incidence [51–53].

The higher incidence of follicular thyroid cancer among non-Hispanic Black children was noteworthy. Follicular thyroid cancer is relatively rare and often associated with an underlying genetic syndrome that predisposes to DTC, such as DICER1, PTEN Hamartoma Syndrome, Carney Complex,) [54,55]. While information on these genetic syndromes was not available in SEER, future research could examine this further. Black patients were more likely to have a larger tumor (>4 cm), but also have a tumor diagnosed as localized, yielding no consistent pattern in terms of disease progression. Larger tumor sizes among Non-Hispanic Asian/Pacific Islander pediatric patients compared to White children after accounting for community-level SES, may suggest more advanced cancer presentation possibly due to other social or structural factors such as barriers to healthcare access; however, additional information would be needed to assess this [56].

We observed some differences in the likelihood of patients receiving a total thyroidectomy. This difference in treatment for example suggested among non-Hispanic Black children may be due to the higher proportion of follicular thyroid carcinoma, which are more likely to yield an indeterminate result on an FNA, which could mean a lobectomy rather than a total thyroidectomy may be the most appropriate course of action, since the lesion may be benign. In addition, it is not clear how patients that undergo two surgeries (e.g., a lobectomy followed by a complete thyroidectomy) are classified in the data.

This study benefits from a large national representative sample for pediatric DTC. Longitudinal data that spans over two decades enabled us to further investigate the temporal trends of pediatric DTC incidence with most recent years. However, there are some limitations that warrant consideration. This registry-based study lacked detailed treatment information, tumor histology data (e.g., PTC subtype or FTC classification), and lifestyle factors, which might be important factors associated with the risk of DTC. SES variables are not individual-level data, hence are subject to misclassification. However, census tract level SES is the finest data resolution so far for registry-based studies and reflect community-level structural characteristics, which are also important health determinants. There might be residual confounding that was not captured by SES indicators in the SEER database. For example, insurance information is important for patients to receive cancer screening and timely treatment, but it is not available in this specific SEER-18 database that we used for analysis.

## Conclusion

Overall pediatric DTC incidence rates declined from 2015 to 2022 (excluding year 2020), but remained increasing among Non-Hispanic Asian/Pacific Islander and those with tumors sizes >4 cm. These findings suggest that the inaugural 2015 ATA guidelines regarding management of thyroid nodules and cancer in children may have led to a decrease in diagnosis of small tumors, suggesting that the pediatric population was previously subject to some over-diagnosis or unnecessary screening. However, we cannot rule out the possibility that the COVID-19 pandemic had a lasting impact on healthcare seeking behaviors after year 2020. Moreover, the continued increase in larger tumor sizes warrants further evaluation of the contribution of environmental and lifestyle risk factors. Some differences in DTC presentation by race were also observed. The greater proportion of non-White racial and ethnic children being diagnosed with larger tumors could be due to inequities related to timely access to care, differential application of thyroid nodule management guidelines, or differences in cancer subtypes. These findings warrant further investigation when additional years of data become available.
